# Diversity of infectious aetiologies of acute undifferentiated febrile illnesses in south and Southeast Asia: a systematic review

**DOI:** 10.1186/s12879-019-4185-y

**Published:** 2019-07-04

**Authors:** Kinley Wangdi, Kaushalya Kasturiaratchi, Susana Vaz Nery, Colleen L. Lau, Darren J. Gray, Archie C. A. Clements

**Affiliations:** 1 Department of Global Health, Research School of Population Health, Australian National University, Action, ACT, Canberra, Australia; 2 Ministry of Health, Colombo, Sri Lanka; 3 Kirby Institute, University of New South Wales, Sydney, NSW Australia; 4 Children’s Health and Environment Program, Child Health Research Centre, The University of Queensland, QLD, South Brisbane, Australia; 5 Faculty of Health Sciences, Curtin University, Perth, WA Australia

**Keywords:** Acute undifferentiated febrile illness, Asia, Infection

## Abstract

**Background:**

Acute undifferentiated febrile illness (AUFI) is caused by a multitude of diverse pathogens, with significant morbidity and mortality in the developing world. The objective of this review was to characterise the diversity and relative importance of common infectious aetiologies of AUFI in South and Southeast Asia.

**Methods:**

We conducted a comprehensive literature review to identify common aetiologies of AUFI in Asian countries. Four medical and life sciences databases including PubMed, Medline, Embase and Cochrane Central, and Google Scholar were searched for articles published from January 1998 to March 2019.

**Results:**

Forty-three studies met the inclusion criteria. Among AUFI cases, viral aetiologies at 18.5% (14888) were more common than bacterial aetiologies (12.9% [10384]). From 80,554 cases, dengue fever was the most common aetiology (11.8%, 9511), followed by leptospirosis (4.4%, 3549), typhoid (4.0%, 3258), scrub typhus (4.0%, 3243) and influenza other than H1N1 (3.1%, 2514). In both adults and children: dengue fever was the leading cause of AUFI with 16.6% (1928) and 18.7% (1281) of the total cases. In admitted patients, dengue fever was the main cause of AUFI at 16.4% (2377), however leptospirosis at 13.9% (2090) was the main cause of AUFI for outpatients. In South Asia, dengue fever was the main cause of AUFI, causing 12.0% (6821) of cases, whereas in Southeast Asia, leptospirosis was the main diagnosis, causing 12.1% (2861) of cases.

**Conclusions:**

In this study the most common causes of AUFI were viral, followed by bacterial and protozoal (malaria) infections. Dengue was the commonest virus that caused AUFI while leptospirosis and typhoid were important bacterial infectious causes. Therefore, it is imperative to maintain a sound epidemiological knowledge of AUFI so that evidence-based diagnostic criteria and treatment guidelines can be developed.

**Electronic supplementary material:**

The online version of this article (10.1186/s12879-019-4185-y) contains supplementary material, which is available to authorized users.

## Background

During the past 20 years, there has been a dramatic emergence and re-emergence of viruses, bacteria and parasitic infections, including novel pathogens as well as those previously believed to be under control. Many of these pathogens cause acute undifferentiated febrile illness (AUFI, or acute febrile illness, AFI). The common causes of AUFI include malaria, dengue fever, enteric fever, leptospirosis, rickettsiosis, hantavirus and Japanese encephalitis [1–3]. AUFI contributes to substantial morbidity and death among children and adults worldwide [4, 5]. Many preventable deaths occur because of incorrect or delayed diagnosis, largely due to limited access to medical care and laboratory diagnostic facilities in the developing countries [6–9]. The majority of patients present with non-specific symptoms such as low-grade fever, general malaise, headache, arthralgia, myalgia, and rash; and usually without a focal point of infection. The symptoms and differential diagnoses of these diseases are similar, making accurate clinical diagnosis difficult without laboratory confirmation [10–12].

In recent decades, dengue has rapidly emerged as a major cause of AUFI in tropical Asia particularly in the World Health Organization (WHO) Southeast Asia (SEA) region [13, 14]. However, many other infectious diseases can cause a dengue-like illness with thrombocytopaenia, including scrub typhus, chikungunya, infectious mononucleosis, malaria, typhoid fever, leptospirosis and acute human immuno-deficiency virus conversion disease [15]. Presumptive diagnosis and reporting of AUFI with thrombocytopaenia as dengue infection would lead to over-reporting of this infection and under-reporting of other illnesses.

Evidence-based decision-making in health requires the availability of sound data, but good quality information on the occurrence of infectious diseases is unavailable for most countries in Asia [16]. The provision of accurate epidemiological data for common pathogens will enable identification of changing patterns of disease aetiology and burden, allowing informed priority setting, and optimal allocation of resources to key areas. Understanding the common causes of AUFI in resource-poor settings in tropical and subtropical countries will help improve case management. In areas where there is limited access to laboratory diagnosis, the local epidemiology of AUFI and validated clinical predictors may help guide presumptive diagnosis and therapeutic interventions. Such information is also crucial for developing appropriate diagnostic tests and guidelines, and informing resource mobilization and public health interventions. Therefore, the objective of this review was to synthesise information on the diversity and relative importance of common infectious aetiologies of AUFI in recent history in South and Southeast Asia given it is a melting point of tropical infectious diseases and a hotspot for disease emergence [14, 17, 18].

## Methods

### Search strategy and inclusion criteria

A systematic literature review was undertaken in four medical and life sciences databases including PubMed, Medline, Embase and Cochrane Central, and Google Scholar search machine was also used. Publications from the last 21 years (January 1998–March 2019) were included because laboratory tests and diseases patterns have changed during recent decades in many parts of South and Southeast Asia. Articles were obtained electronically or in paper form. The search words included: i) aetiology OR etiology OR causes AND ii) acute febrile illnesses OR iii) undifferentiated fevers AND Asia OR Thailand OR Malaysia OR Singapore OR India OR Sri Lanka OR Nepal OR Bangladesh OR Pakistan OR Vietnam OR Laos OR Cambodia OR Indonesia OR Myanmar OR Timor-Leste OR Bhutan OR Maldives OR Philippines. The review included articles published in English only.

We did not limit our search by study design or patient age. Data were derived from studies on inpatients as well as outpatients with AUFI with no focus of infections identified after taking a detailed history and clinical examination. Inclusion criteria were: a) primary articles, published in peer review journals on AFI/AUFI in South Asia (Bhutan, Bangladesh, India, Nepal, and Sri Lanka) and Southeast Asia (Cambodia, Laos, Indonesia, Malaysia, Myanmar, Philippines, Singapore, Thailand, and Vietnam); b) published reports between January 1998 and March 2019 (to improve the reliability of laboratory confirmation and to reflect the distribution of more recent disease patterns) and c) published in English. Exclusion criteria included: a) studies carried out in other parts of Asia (Middle East and central Asia); b) studies conducted before 1998; c) articles such as preliminary reports, and case reports; d) editorials, opinions, review articles, vaccine and drug trials; and e) case reports and fever associated with a travel history (Additional file 1: Table S1)*.* Titles and abstracts were screened for compliance with the inclusion criteria and then full papers were reviewed.

### Data analysis

The selection of citations by title and abstract was carried out independently by two researchers (KW and SKK). The selected studies underwent a full-text review for all potentially relevant studies. Data from the 43 included studies were independently extracted in a spreadsheet by KW and SKK. Information from each paper was extracted and entered in to a Microsoft Excel (2010 version) spread sheet. Descriptive data included study location, study period, type of patients (inpatients/ outpatients/ both), age range and duration of fever. Quantitative data recorded included number of patients, pathogens isolated, and common presenting signs and symptoms. Paediatric data were defined as those that included patients younger than 16 years. Studies with non-segregated data for adults and children were analysed separately. Data for pathogens isolated in each study were compiled and analysed in aggregate to compare common aetiologies of AUFI. The proportion of fevers confirmed through laboratory diagnosis in each study were recorded as the main outcome measure.

### Risk of bias assessment

The risk of bias (ROB) of the included studies was assessed using a modified checklist used previously [19]. The studies were assessed using eight questions with a possible maximum count of eight safe-guards (Additional file 1: Table S2), with three questions to assess external validity, and five questions for internal validity. We did not assess the ROB for the sampling methodology of populations with acute febrile illness, as these were defined populations presenting to a health facility with acute infection and no population-based sampling was used to capture these populations.

## Results

### Identification of studies

**Fig. 1:**
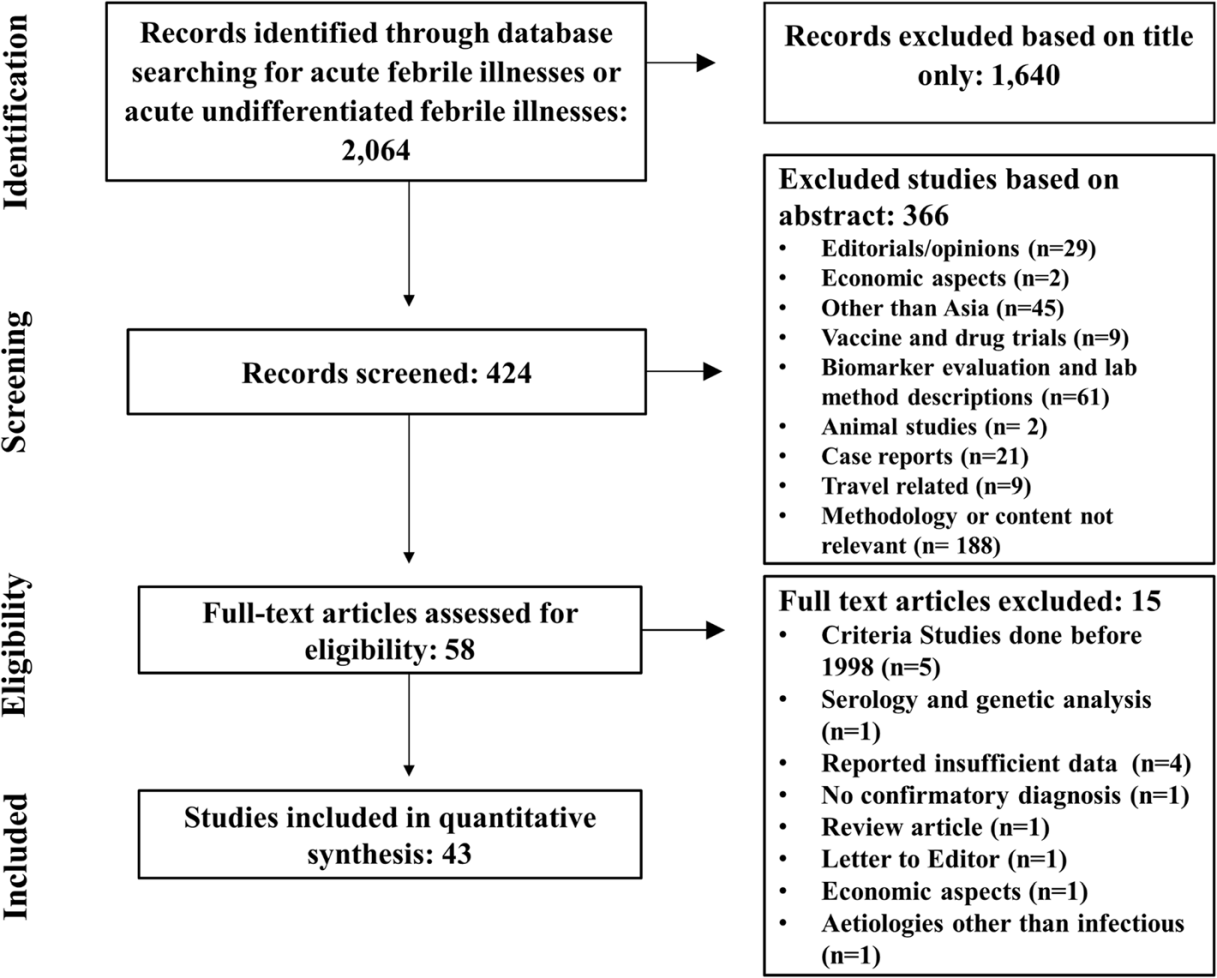
Study selection

Using the key words in the search, 2064 articles were identified from four life science data base (PubMed, Medline, Web of Science, Embase) and Google Scholar. The titles and abstracts of all studies identified by the search strategy were screened for their relevance to this review and 1640 records that were not relevant for fever with infectious aetiologies were discarded. Four hundred fifteen (424) reports were screened further and from those records, 366 were excluded after reviewing the abstracts as they did not meet the inclusion criteria. All remaining 58 full text articles were reviewed using pre-determined criteria. A full-text review led to the exclusion of a further 15 papers, including five studies with potentially relevant data that were excluded as three of them were carried out in 1991, one study in 1994 and the other study from 1994 to 1999. The remaining 43 studies were from 11 countries in South and Southeast Asia, including a multicentre study, were then analysed (Fig. 1**)**.

### Characteristics of included studies

**Fig. 2:**
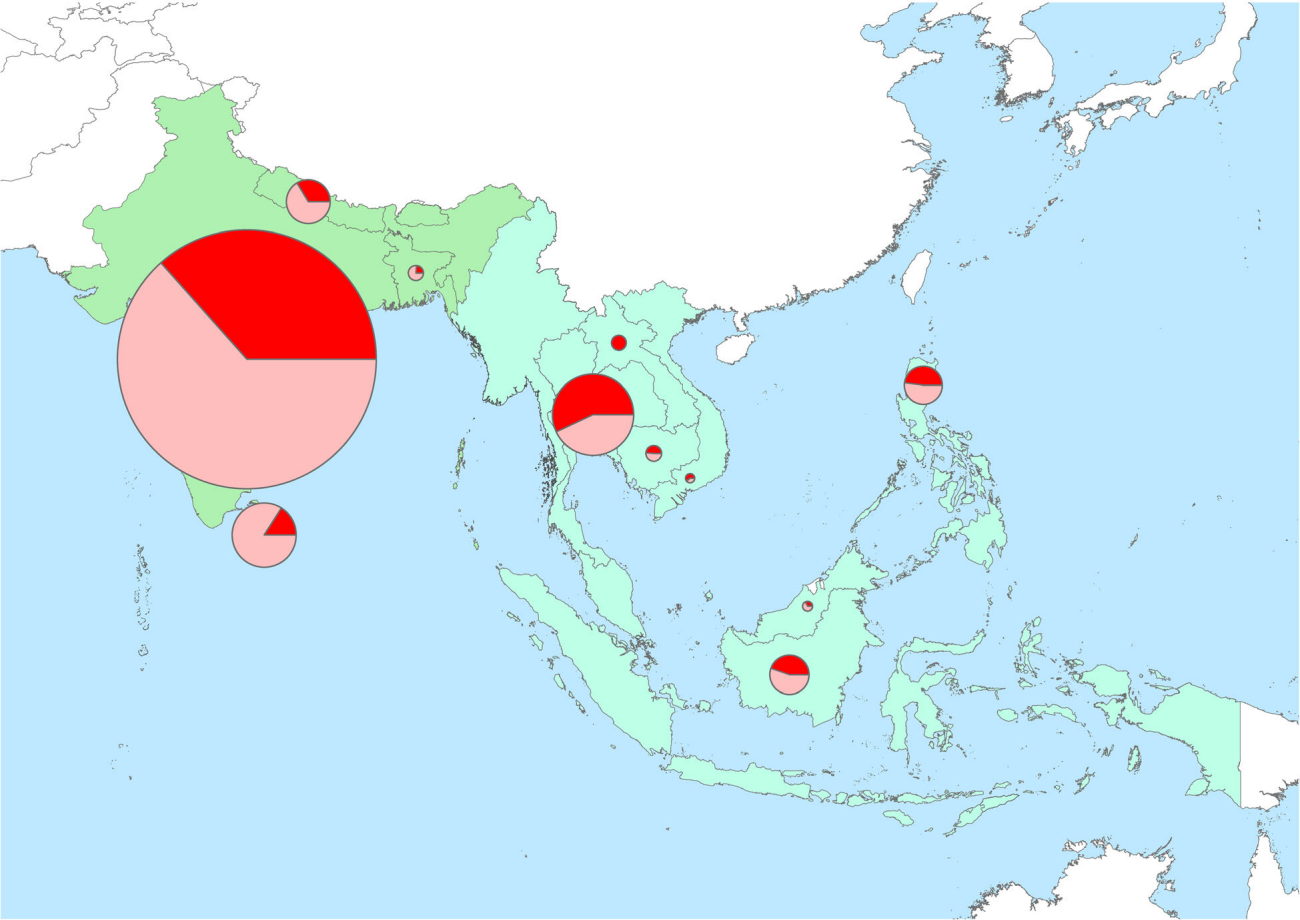
Summary of the studies by countries and regions. The size of the circles indicates the relative number of patients and light colour is the proportion of study subjects with unidentified aetiology. Countries with blue colour are in South East Asia and green colour are in South Asia

Twenty-eight studies were from South Asia, of which 20 were from India. In the South East Asia, there were 15 studies and Thailand reported the highest number of studies in the region with nine studies **(**Fig. 2). There was one multi-centre study carried out in Indonesia, Malaysia, Philippines, Thailand and Vietnam. Most were prospective studies (*n* = 31) and six studies were retrospective; three were cross-sectional; one cohort study and two active fever surveillance (Table 1).

**Table 1:** *Summary of studies included in the analysis*

First author, year and reference	Country	Design study	Study duration	In patients/ outpatients	Number of patients	Age range	Adult/Children only	Sex	Duration of fever (mean duration)
Abhilash et al., 2016 [20]	India	Prospective observational study	12 months	OPD + ED	1258	> 15 years	Adults + children	M = 680 F = 568	3–14 days
Ahmad et al., 2016 [44]	India	Retrospective observational study	12 months	IP	298	> 2 years	Adults + children	Not specified	AUFI
Andrews et al., 2014 [45]	India	Retrospective and Patient admitted prospective study	2 months	IP	369	> 13 years	Adults	F = 118 M = 251	AUFI
Arora et al., 2014 [46]	India	Retrospective study	24 months	IP + OPD	38,635	All ages groups	Adult + Children	Not specified	AUF
Capeding et al., 2013 [11]	Indonesia, Malaysia, Philippines, Thailand and Vietnam	Active fever surveillance, cohort study	9.8 months	Community based	289	2–14 years	Children	Not specified	< 14 days
Chheng et al., 2013 [42]	Cambodia	Prospective study	12 months	IP	1225	< 16 years	Children	M = 668 F = 557	< 28 days
Chikkaveerariah et al., 2016 [47]	India	Prospective observational study	24 months	IP	150	> 14 years	Adults	F = 69 M = 81	AUFI
Chrispal et al., 2010 [1]	India	Prospective observational study	12 months	IP	398	> 16 years Mean 39.5	Adults	M = 239 F = 159	5–21 days
Das et al., 2015 [48]	India	Cross-sectional study	6 months	IP	205	All ages	Adults + Children	F = 89 M = 116	Acute febrile illness
Ellis et al., 2006 [49]	Thailand	Prospective study	33 months	IP + OPD	613	20–87 years	Adults	M = 325 F = 288	Fever over previous 48 h and fever longer than 48 h cause of fever not yet known
Gopalakrishnan et al., 2013 [21]	India	Prospective observational	18 months	IP	403	> 16 years	Adults	M = 264 F = 139	5–14 days
Joshi et al., 2008 [22]	India	Retrospective review of electronic discharge summaries	6 months	IP	1197	> 12 years	Adults	M = 640 F = 557	< 14 days
Kammili et al., 2013 [23]	India	Prospective descriptive hospital based study	2 months	IP	100	All ages	Both children and adults	Not specified	> 24 h
Kashinkunti et al., 2013 [37]	India	Prospective observational	Not specified	IP	100	> 16 years	Adults	M = 58 F = 42	< 15 days
Kasper et al., 2012 [24]	Cambodia	Fever surveillance study	Not specified	OPD	9997	> 2 years Mean 19.6 Median 16.9	Adults and children	M = 5398 F = 4599	< 10 days
Kumar et al., 2008 [39]	India	Prospective study	12 months	OPD	298	6 months −12 years	Children	M = 117 F = 181	< 15 days
Laoprasopwattana et al., 2012 [25]	Thailand	Prospective cohort study	4 months	IP + OPD	50	1 month −15 years	Children	Not specified	< 7 days
Leelarasamee et at., 2004 [2]	Thailand	Prospective epidemiological study	36 months	OPD	1137	> 2 years	Adults + Children	Not specified	< 1 day
Mayxay et al., 2013 [26]	Laos	Prospective study	30 months	IP + OPD	1938	5–49 median 19	Adults/children	M = 1124 F = 814	< 8 days
McGready et al., 2010 [50]	Thailand	Prospective cohort study	28 months	IP	409	> 15 years	Pregnant females only	M = 467 F = 409	Any fever
Mittal et al., 2015 [51]	India	Retrospective observational study	12 months	IP + OPD	2547	> 18 years	Adults	F = 884 M = 1663	AUFI
Murdoch et al., 2004 [36]	Nepal	Prospective study	3 months	IP + OPD	876	> 14 years Median 27	Adults	F = 409	24 h
Oishr et al., 2006 [27]	Philippines	Prospective study	24 months	IP	503	2–17 years	Children	M = 298 F = 205	< 5 days
Phuong et al., 2006 [28]	Vietnam	Prospective study	12 months	OPD	2096	All ages	Both children and adults	M = 1229 F = 865	< 14 days
Pradutkanchana et al., 2003 [7]	Thailand	Prospective study	1 month	IP	180	Less than 15 years	Children	Not specified	< 21 days
Punjabi et al., 2012 [43]	Indonesia	Prospective study	27 months	IP	226	1–80 years	Adults and children	M = 127 F = 99	1–30 days
Rafizah et al., 2012 [52]	Malaysia	Hospital-based cross sectional study	6 months	IP	999	> 18 years	Adults	F = 543 M = 456	Acute fever
Rani et al., 2016 [53]	India	Retrospective study	6 Months	IP + OPD	200	All ages	Adults + children	F = 82 M = 118	Acute febrile illness
Ray et al., 2012 [29]	India	Prospective descriptive study	12 months	IP + OPD	540	All age groups	Both children and adults	M = 329 F = 211	< 7 days
Reller et al., 2011 [31]	Sri Lanka	Prospective study	8 months	IP + OPD	773	> 2 years	Both children and adults	M = 463 F = 310	< 7 days
Reller et al., 2012 [30]	Sri Lanka	Prospective study	8 months	IP + OPD	859	> 2 years	Both children and adults	M = 526 F = 333	< 7 days
Sabchareon et al., 2012 [54]	Thailand	Prospective cohort study	48 months	Community based + IP + OPD	3401	3–15 years	Children	M = 1733 F = 1668	All documented fever with school absenteeism
Suttinont et al., 2006 [12]	Thailand	Prospective observational study in 5 hospitals	12 months	IP	845	> 15 years Median 38	Adults	M = 661 F = 184	< 15 days
Thompson et al., 2015 [56]	Nepal	Prospective study	38 months	IP + OPD	627	> 2 years	Adults + children	Not specified	UFI
Zaki et al., 2010 [41]	India	Prospective observational study	4 months	IP	602	1 month −12 years	Children	Not specified	< 21 days
Kingston et al., 2018 [38]	Bangladesh	Prospective study	12 months	IP	416	≥12 years	Adults	Not specified	< 21 days
Raina et al., 2018 [32]	India	Cohort study	2 months	IP	1164	> 18 years	Adults	Not specified	≤14 days
Shelke et al., 2017 [33]	India	Prospective cross-sectional	18 months	IP	270	All ages	Adults + children	M = 138 F = 132	< 14 days
Gautam et al., 2019 [57]	Nepal	Cross-sectional study	12 months	IP	1585	> 1 year	Adult + children	M = 728 F = 757	> 4 days
Bodinayake et al., 2018 [35]	Sri Lanka	Prospective study	12 months	IP	976	≥1 years	Adults + children	M = 628 F = 348	≤3 days
Salagre et al., 2017 [55]	India	Prospective observational study	2 months	IP	276	> 13 years	Adults	M = 187 F = 89	AFI
Andrews et al., 2018 [34]	India	Prospective observational study	12 months	IP	1324	> 13 years	Adults	M = 837 F = 487	< 14 days
Wangrangsimakul et al., 2018 [40]	Thailand	Prospective observational study	27 months	IP	200	≥15 years	Adults	M = 114 F = 86	< 21 days

*ED* Emergency department, *IP* Inpatients, *OPD* Outpatient department, *M* Males, *F* Females

Definition of fever duration in the acute febrile illnesses described in these studies varied widely, from 1 to 30 days. Of the 43 included studies, 19 included duration of fever of < 14 days [2, 11, 20–36], seven studies had fever duration of < 21 days [7, 12, 37–41], three studies reported fever with the duration of < 30 days [1, 42, 43], 13 studies did not define any specific duration [44–56] and one study recruited patients with acute fever of more than 4 days [57]. Similarly, the temperature threshold used to define fever varied from 37.5–38.5°Celsius. Sixteen studies included adult (> 16 years) patients [1, 12, 21, 22, 32, 36–38, 40, 45, 47, 49–52, 55], eight studies included children (< 16 years) [7, 11, 25, 27, 39, 41, 42, 54] and 19 studies included patients of all ages [2, 20, 23, 24, 26, 28–31, 33–35, 43, 44, 46, 48, 53, 56, 57]. The number of patients involved in each study varied from 50 to 38,635. Data on a total of 80,554 patients were included in the analysis of which 14.4% (11706) were adults, 8.5% (7840) were children and 77.1% (62068) were not specified either as adults or children and 39 cases could not be assigned to any age groups [35]. Among adults, mean age varied from 27 to 39.5 years and among children from 2 to 9.5 years. There were 19,030 males, 14,625 females (with a male to female ratio of 1.3:1), and gender was not reported for 46,899 patients.

Twenty-five studies included patients admitted to hospital [1, 7, 12, 21–23, 27, 32–35, 37, 38, 40–45, 47, 48, 50, 52, 55, 57] making up 18.7% (14420) of the total study sample (80554). Six studies included patients attending outpatients department corresponding to 18.7% (15075) of study sample [2, 11, 20, 24, 28, 39]. Twelve studies included patients attending outpatient departments (OPD) and those admitted to hospital wards representing 63.4% (51059) of total study sample [25, 26, 29–31, 36, 46, 49, 51, 53, 54, 56]. Common presenting symptoms were given in 24 studies, corresponding to 30,397 patients. Amongst these 23 studies, the most common presenting symptom was headache 39.7% (12072) followed by cough 29.7% (9035) and chills 20.5% (6241) (Additional file 1: Table S3).

**Fig. 3:**
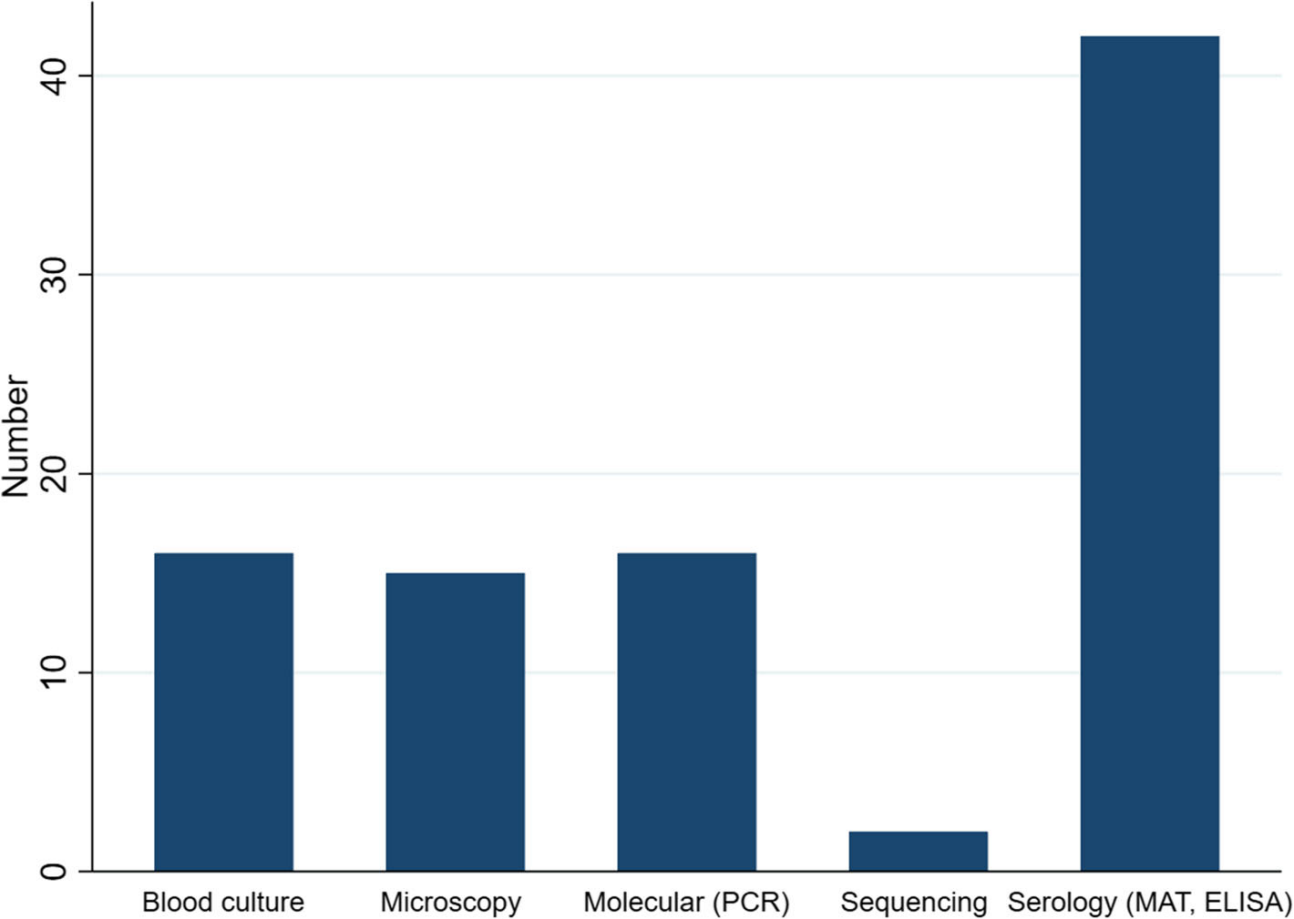
Summary of different diagnostic methods. (MAT- microscopic agglutination test; ELISA- enzyme-linked immunosorbent assay; PCR- polymerase chain reaction)

In all studies excepting one [34], diagnoses were made according to interpretation of antibody titres. Pathogen-specific IgM titres were determined by using IgM-capture enzyme-linked immunosorbent assay (ELISA) kits, which are commercially available. Molecular testing (using polymerase chain reaction [PCR] was carried out in 16 studies [24–27, 29, 30, 35, 38–40, 42, 49, 50, 54–56]. Serological diagnoses were confirmed by blood cultures in 16 studies out of 43 [2, 20–22, 24, 26, 32, 37, 40, 42, 43, 45, 47, 48, 50, 51]. Microscopy was used for the diagnosis in 15 studies [20–22, 24, 26, 32, 37, 43, 44, 46, 47, 49–51, 55]**.** Nucleotide sequencing was done in two studies [29, 35] (Fig. 3 and Additional file 1: Table S4).

Aetiology of AUFI was identified in 37.7% (30333) of patients: with viral aetiologies in 18.5% (14888) being the most common, followed by bacterial and protozoal aetiologies with 12.9% (10384), and 2.8% (2281) respectively. The underlying diagnosis could not be ascertained in 64.6% of patients (52003). Twenty studies reported 378 deaths in patients with AUFI [1, 2, 20–22, 25–27, 31, 34, 36, 38, 41–45, 48–50]. Co-infections were reported in 1.2% (981) of total cases, the most common co-infections with two organisms 0.9% (740).

### Aetiology of AUFI by age group

**Fig. 4:**
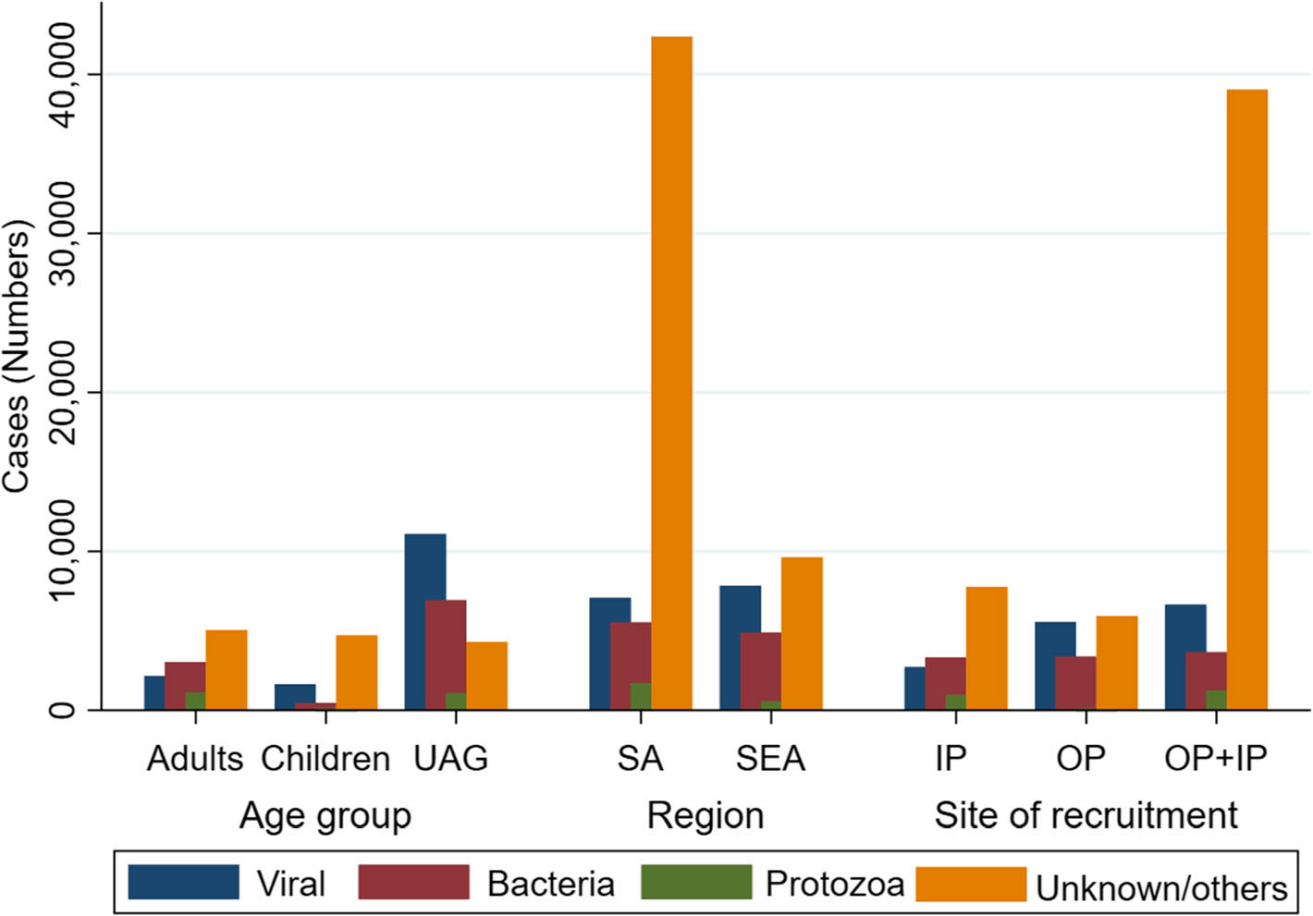
Summary graph of main categories of AUFI across age group, region and site of patient recruitment in Asia. (UAG- unknown age group, IP-inpatient; OP-outpatient; SEA-Southeast Asia, SA- South Asia)

In adults, the commonest infection was from bacterial causes at 26.1% (3037), followed by viral aetiologies 18.6% (2169). The most common aetiologies of AUFI in adults were dengue fever 16.6% (1928), scrub typhus 10.7% (1244), malaria 9.8% (1139), leptospirosis 6.3% (732) and typhoid 6.0% (696). On the other hand, viral infection was the commonest cause of fever among children corresponding to 23.8% (1625) of the diagnosed cases, followed by bacterial aetiologies and malaria (corresponding to 6.4% (435) and 0.8% (57) of diagnosed cases, respectively). Dengue fever, chikungunya, and typhoid were the commonest cause of AUFI in children representing 18.7% (1281), 1.7% (114), and 1.6% (107) respectively. In the unspecified age group (UAG), dengue fever was the commonest cause of AUFI with 10.2% (6302) of total cases; leptospirosis was the second commonest cause with 4.4% (2729); typhoid and malaria contributed 4.0% (2455) and 1.7% (1085) of total cases (Table 2 and Fig. 4).

**Table 2:** Common aetiologies of AUFI stratified by age

Organism	Adults (*n*; %)	Children (*n*; %)	UAG (*n*; %)	Total^⁑^ (*n*; %)
**Viral aetiologies**	**2169 (18.6)**	**1625 (23.8)**	**11,094 (17.9)**	**14,888 (18.5)**
Dengue	1928 (16.6)	1281 (18.7)	6302 (10.2)	9511 (11.8)
JE^***^	5 (0.0)	71 (1.0)	233 (0.4)	309 (0.4)
Influenza^**^	180 (1.5)	48 (0.7)	2286 (3.7)	2514 (3.1)
H1N1	5 (0.0)	1 (0.0)	513 (0.8)	519 (0.6)
Chikungunya	15 (0.1)	114 (1.7)	326 (0.5)	455 (0.6)
Hepatitis A	8 (0.1)	7 (0.1)	58 (0.1)	73 (0.1)
Hepatitis B	5 (0.0)	0 (0.0)	267 (0.4)	272 (0.3)
Hepatitis E	2 (0.0)	0 (0.0)	1038 (1.7)	1040 (1.3)
Flavi virus	0 (0.0)	65 (1.0)	0 (0.0)	65 (0.1)
Para influenza 1	0 (0.0)	10 (0.1)	0 (0.0)	10 (0.0)
Para influenza 3	0 (0.0)	28 (0.4)	0 (0.0)	28 (0.0)
Hanta virus	2 (0.0)	0 (0.0)	71 (0.1)	73 (0.1)
HIV	19 (0.2)	0 (0.0)	0 (0.0)	19 (0.0)
**Bacterial aetiologies**	**3037 (26.1)**	**435 (6.4)**	**6912 (11.1)**	**10,384 (12.9)**
Leptospirosis	732 (6.3)	88 (1.3)	2729 (4.4)	3549 (4.4)
Typhoid	696 (6.0)	107 (1.6)	2455 (4.0)	3258 (4.0)
Paratyphoid	57 (0.5)	0 (0.0)	0 (0.0)	57 (0.1)
Rickettsiosis diseases	1449 (12.5)	140 (2.0)	1654 (2.7)	3243 (4.0)
Scrub typhus	1244 (10.7)	103 (1.5)	1512 (2.4)	2859 (3.5)
Murine typhus	171 (1.5)	0 (0.0)	101 (0.2)	272 (0.3)
Spotted fever	34 (0.3)	37 (0.5)	41 (0.1)	112 (0.1)
Q fever	7 (0.1)	0 (0.0)	0 (0.0)	7 (0.0)
E coli	11 (0.1)	21 (0.3)	26 (0.0)	58 (0.1)
Burkholderia pseudomallei	3 (0.0)	14 (0.2)	6 (0.0)	23 (0.0)
Tuberculosis	29 (0.2)	6 (0.1)	8 (0.0)	43 (0.1)
*Klebsiella pneumoniae*	1 (0.0)	0 (0.0)	2 (0.0)	3 (0.0)
Haemophilus influenza	0 (0.0)	0 (0.0)	9 (0.0)	9 (0.0)
Staph aureus	0 (0.0)	37 (0.5)	12 (0.0)	49 (0.1)
Strep pneumoniae	51 (0.4)	18 (0.3)	6 (0.0)	75 (0.1)
Strep Gr A	0 (0.0)	0 (0.0)	2 (0.0)	2 (0.0)
Strep Gr C	0 (0.0)	0 (0.0)	1 (0.0)	1 (0.0)
Neisseria meningitis	1 (0.0)	4 (0.1)	2 (0.0)	7 (0.0)
**Protozoa**	**1139 (9.8)**	**57 (0.8)**	**1085 (1.7)**	**2281 (2.8)**
Malaria	1139 (9.8)	57 (0.8)	1085 (1.7)	2281 (2.8)
**Fungal aetiologies**	**0 (0.0)**	**0 (0.0)**	**3 (0.0)**	**3 (0.0)**
Yeast non-Cryptococci	0 (0.0)	0 (0.0)	2 (0.0)	2 (0.0)
*Cryptococcus neoformans*	0 (0.0)	0 (0.0)	1 (0.0)	1 (0.0)
**Co infections**	**251 (2.2)**	**30 (0.4)**	**700 (1.1)**	**981 (1.2)**
Co infection^*^	226 (1.9)	30 (0.4)	484 (0.8)	740 (0.9)
Co infection^†^	25 (0.2)	0 (0.0)	210 (0.3)	235 (0.3)
Co infection^‡^	0 (0.0)	0 (0.0)	6 (0.0)	6 (0.0)
**Unknown/others**	**5036 (43.3)**	**4693 (68.6)**	**42,274 (68.1)**	**52,003 (64.6)**
**Deaths**	**125 (33.1)**	**81 (21.4)**	**172 (45.5)**	**378 (100.0)**

UAG Unknown age group, ^***^JE Japanese B Encephalitis; ^⁑^39 cases in the manuscript could not be assigned to any age group; ^**^influenza other than H1N1; ^*^co-infection with two organisms; ^†^co-infection with three organisms; ^‡^co-infection with more than three organisms

The bold face shows the cumulative number of the stratified groups

### Aetiology of AUFI by site of patient recruitment

Among the 14,450 hospitalised patients, bacterial infection 23.1% (3340) was the leading cause of fever. However, the most common aetiology of AUFI was dengue fever 16.4% (2377), followed by scrub typhus 10.0% (1449), malaria 6.9% (990), and leptospirosis 6.8% (989). A total of 7053 representing 48.8% did not have a known diagnosis. Even though viral infections (36.7%, 5536) were the main cause for fever in outpatients, leptospirosis 13.9% (2090) was the commonest cause of AUFI followed by influenza other than HINI 13.8% (2077), dengue 8.5% (1277), and hepatitis E 6.9% (1038). Dengue was the commonest infection in patients recruited from both IP and OPD 11.5% (5857), followed by typhoid 3.8% (1940), malaria 2.4% (1234), scrub typhus 2.3% (1165), and leptospirosis 0.9% (470) respectively **(**Table 3 and Fig. 4).

**Table 3:** Aetiology of AUFI by site of patient recruitment

Organism	IP (*n*; %)	OP (*n*; %)	OP+IP (*n*; %)	Total (n; %)
**Viral aetiologies**	**2721 (18.8)**	**5536 (36.7)**	**6631 (13.0)**	**14,888 (18.5)**
Dengue	2377 (16.4)	1277 (8.5)	5857 (11.5)	9511 (11.8)
JE^***^	77 (0.5)	7 (0.0)	225 (0.4)	309 (0.4)
Influenza^**^	86 (0.6)	2077 (13.8)	351 (0.7)	2514 (3.1)
HINI	6 (0.0)	513 (3.4)	0 (0.0)	519 (0.6)
Chikungunya	54 (0.4)	224 (1.5)	177 (0.3)	455 (0.6)
Hepatitis A	0 (0.0)	62 (0.4)	11 (0.0)	73 (0.1)
Hepatitis B	4 (0.0)	267 (1.8)	1 (0.0)	272 (0.3)
Hepatitis E	2 (0.0)	1038 (6.9)	0 (0.0)	1040 (1.3)
Flavi virus	65 (0.4)	0 (0.0)	0 (0.0)	65 (0.1)
Para influenza 1	10 (0.1)	0 (0.0)	0 (0.0)	10 (0.0)
Para influenza 3	28 (0.2)	0 (0.0)	0 (0.0)	28 (0.0)
Hanta virus	1 (0.0)	71 (0.5)	1 (0.0)	73 (0.1)
HIV	11 (0.1)	0 (0.0)	8 (0.0)	19 (0.0)
**Bacterial aetiologies**	**3340 (23.1)**	**3383 (22.4)**	**3662 (7.2)**	**10,385 (12.9)**
Leptospirosis	989 (6.8)	2090 (13.9)	470 (0.9)	3549 (4.4)
Typhoid	406 (2.8)	912 (6.0)	1940 (3.8)	3258 (4.0)
Paratyphoid	57 (0.4)	0 (0.0)	0 (0.0)	57 (0.1)
Rickettsial diseases	1670 (11.6)	341 (2.3)	1232 (2.4)	3243 (4.0)
Scrub typhus	1449 (10.0)	245 (1.6)	1165 (2.3)	2859 (3.5)
Murine typhus	178 (1.2)	65 (0.4)	29 (0.1)	272 (0.3)
Spotted fever	43 (0.3)	31 (0.2)	38 (0.1)	112 (0.1)
Q fever	7 (0.0)	0 (0.0)	0 (0.0)	7 (0.0)
E coli	29 (0.2)	25 (0.2)	4 (0.0)	58 (0.1)
Burkholderia pseudomallei	17 (0.1)	3 (0.0)	3 (0.0)	23 (0.0)
Tuberculosis	36 (0.2)	0 (0.0)	7 (0.0)	43 (0.1)
Klebsiella pneumonia	2 (0.0)	0 (0.0)	2 (0.0)	4 (0.0)
Haemophilus influenza	9 (0.1)	0 (0.0)	0 (0.0)	9 (0.0)
Staph aureus	39 (0.3)	8 (0.1)	2 (0.0)	49 (0.1)
Strep pneumonia	73 (0.5)	2 (0.0)	0 (0.0)	75 (0.1)
Strep Gr A	1 (0.0)	0 (0.0)	1 (0.0)	2 (0.0)
Strep Gr C	0 (0.0)	0 (0.0)	1 (0.0)	1 (0.0)
Neisseria meningitis	5 (0.0)	2 (0.0)	0 (0.0)	7 (0.0)
**Protozoa**	**990 (6.9)**	**57 (0.4)**	**1234 (2.4)**	**2281 (2.8)**
Malaria	990 (6.9)	57 (0.4)	1234 (2.4)	2281 (2.8)
**Fungal aetiologies**	**3 (0.0)**	**0 (0.0)**	**0 (0.0)**	**3 (0.0)**
Yeast non-Cryptococci	2 (0.0)	0 (0.0)	0 (0.0)	2 (0.0)
Cryptococcus neoformans	1 (0.0)	0 (0.0)	0 (0.0)	1 (0.0)
**Co infections**	**343 (2.4)**	**176 (1.2)**	**496 (1.0)**	**1015 (1.3)**
Co infection^*^	331 (2.3)	169 (1.1)	270 (0.5)	770 (1.0)
Co infection^†^	12 (0.1)	7 (0.0)	220 (0.4)	239 (0.3)
Co infection^‡^	0 (0.0)	0 (0.0)	6 (0.0)	6 (0.0)
**Unknown**	**7053 (48.8)**	**5923 (39.3)**	**39,036 (76.5)**	**52,012 (64.5)**
**Deaths**	**270 (71.4)**	**30 (7.9)**	**78 (20.6)**	**378 (100.0)**

IP Inpatients, OP Outpatients; ^***^JE- Japanese B Encephalitis, HAV Hepatitis A virus; HBV Hepatitis E virus, HEV Hepatitis E virus,

^**^Influenza other than H1N1; ^*^co-infection with two organisms; ^†^co-infection with three organisms; ^‡^co-infection with more than three organisms

The bold face shows the cumulative number of the stratified groups

### Aetiology of AUFI by region

In both the regions, viral aetiologies were the leading cause of AUFI with 33.0% (7828) and 12.4% (7060) for SEA and South Asia, respectively. However, there was significant differences in the burden of AUFI when stratified by individual aetiologies. In South Asia, the commonest cause of fever was dengue fever 12.0% (6821) followed by typhoid 4.3% (2449), and malaria 3.0% (1722). While Leptospirosis was the leading infection 12.1% (2861) in SEA followed by dengue fever 11.4% (2690), influenza other than H1N1 10.6% (2511), and hepatitis E 4.4% (1038) (Table 4 and Fig. 4).

**Table 4:** Aetiology by region (Southeast Asia and South Asia)

Organism	SEA (*n*; %)	South Asia (*n*; %)	Total (*n*; %)
**Viral aetiologies**	**7828 (33.0)**	**7060 (12.4)**	**14,888 (18.5)**
Dengue	2690 (11.4)	6821 (12.0)	9511 (11.8)
JE^***^	309 (1.3)	0 (0.0)	309 (0.4)
Influenza^**^	2511 (10.6)	3 (0.0)	2514 (3.1)
HINI	514 (2.2)	5 (0.0)	519 (0.6)
Chikungunya	256 (1.1)	199 (0.3)	455 (0.6)
Hepatitis A	62 (0.3)	11 (0.0)	73 (0.1)
Hepatitis B	267 (1.1)	5 (0.0)	272 (0.3)
Hepatitis E	1038 (4.4)	2 (0.0)	1040 (1.3)
Flavi virus	65 (0.3)	0 (0.0)	65 (0.1)
Para influenza 1	10 (0.0)	0 (0.0)	10 (0.0)
Para influenza 3	28 (0.1)	0 (0.0)	28 (0.0)
Hanta virus	71 (0.3)	2 (0.0)	73 (0.1)
HIV	7 (0.0)	12 (0.0)	19 (0.0)
**Bacterial aetiologies**	**4873 (20.6)**	**5512 (9.7)**	**10,385 (12.9)**
Leptospirosis	2861 (12.1)	688 (1.2)	3549 (4.4)
Typhoid	809 (3.4)	2449 (4.3)	3258 (4.0)
Paratyphoid	0 (0.0)	57 (0.1)	57 (0.1)
Rickettsial diseases	1009 (4.3)	2234 (3.9)	3243 (4.0)
Scrub typhus	764 (3.2)	2095 (3.7)	2859 (3.5)
Murine typhus	146 (0.6)	126 (0.2)	272 (0.3)
Spotted fever	99 (0.4)	13 (0.0)	112 (0.1)
Q fever	7 (0.0)	0 (0.0)	7 (0.0)
E coli	49 (0.2)	9 (0.0)	58 (0.1)
Burkholderia pseudomallei	23 (0.1)	0 (0.0)	23 (0.0)
Tuberculosis	21 (0.1)	22 (0.0)	43 (0.1)
Klebsiella pneumoniae	3 (0.0)	1 (0.0)	4 (0.0)
Haemophilus influenza	9 (0.0)	0 (0.0)	9 (0.0)
Staph aureus	49 (0.2)	0 (0.0)	49 (0.1)
Strep pneumoniae	24 (0.1)	51 (0.1)	75 (0.1)
Strep Gr A	2 (0.0)	0 (0.0)	2 (0.0)
Strep Gr C	1 (0.0)	0 (0.0)	1 (0.0)
Neisseria meningitides	6 (0.0)	1 (0.0)	7 (0.0)
**Protozoa**	**559 (2.4)**	**1722 (3.0)**	**2281 (2.8)**
Malaria	559 (2.4)	1722 (3.0)	2281 (2.8)
Fungal aetiologies	**3 (0.0)**	**0 (0.0)**	**3 (0.0)**
Yeast non Cryptococci	2 (0.0)	0 (0.0)	2 (0.0)
Cryptococcus neoformans	1 (0.0)	0 (0.0)	1 (0.0)
**Co infections**	**815 (3.4)**	**196 (0.3)**	**1011 (1.3)**
Co infection^*^	592 (2.5)	178 (0.3)	770 (1.0)
Co infection^†^	217 (0.9)	18 (0.0)	235 (0.3)
Co infection^‡^	6 (0.0)	0 (0.0)	6 (0.0)
**Unknown**	**9621 (40.6)**	**42,389 (74.5)**	**52,010 (64.5)**
**Deaths**	**114 (30.2)**	**264 (69.8)**	**378 (100.0)**

South Asian countries included: India, Bhutan, Bangladesh, Sri Lanka, and Nepal

Southeast Asian (SEA) countries included: Thailand, Indonesia, Malaysia, Laos, Philippines, Cambodia and Vietnam ^***^JE- Japanese B encephalitis;

^**^influenza other than H1N1; ^*^co-infection with two organisms; ^†^co-infection with three organisms; ^‡^co-infection with more than three organisms

The bold face shows the cumulative number of the stratified groups

### Case fatalities

A total of 378 deaths were reported across 20 studies corresponding to a case fatality rate (CFR) of 0.5%. There were 114 deaths in the SEA region with a CFR of 0.5%. In South Asia, the CFR was 0.5% with 264 deaths. More than half (172) of the deaths were in patents whose age was unknown, with a case fatality of 0.3%, followed by children with 81 deaths (CFR of 1.2%), and adults with 112 deaths (CFR 1.3%). Most of the deaths occurred in hospitalised patients 270 (CFR 1.9%) followed by both inpatients and outpatients 78 (CFR 0.2%).

### Risk of bias

The quality of the studies including types of study, randomization and other characteristics was assessed through eight safeguards against bias as outlined in the Additional file 1: Table S2. The ranges of score were 4–8. The most common safeguard missing was study’s target population. Only 15 studies recruited patients of all ages presenting with AUFI. The other studies restricted study population either to children or adults. All studies had study instrument that had validity and reliability (Additional file 1: Table S5).

## Discussion

The findings of this review illustrate that in tropical and subtropical South and Southeast Asian countries, the most common causes of AUFI were viruses, followed by bacteria and malaria. Generally, dengue fever was the commonest cause followed by leptospirosis and typhoid. Consistent with our findings, the decline in malaria cases in Asia and Africa has resulted in a relative increase in non-malarial AUFIs in these continents [58]. Non-malarial fever was responsible for 20–50% of all fevers in Asia and Africa in children over 5 years of age and adults [59]. While dengue was mostly frequently reported febrile illness in Latin America [60].

Leptospirosis was the leading cause of AUFI in the Southeast region similar to other reported studies from that region [61–65], in agricultural workers [66, 67] and mostly in males [68]. The ability of all countries in the region to accurately report and monitor leptospirosis hinges strongly on their respective capacity to provide accurate and reliable laboratory diagnosis, and robust reporting and surveillance systems [69, 70]. While the microscopic agglutination test (MAT) is considered to be the gold standard serological test [71], there are limitations to the test including a need for live cultures of *Leptospira* of different serogroups, cross-reactions between serogroups and serovars, poor sensitivity in the first week of illness, and persistence of high titres for many years after an infection. Conversely, treatment with antibiotics can blunt the immune response in leptospirosis, reducing the number of cases detectable by serology [72]. Hence, the number of leptospirosis patients reported in this review could be under or overestimated.

Dengue was the commonest cause of AUFI in South Asia contrary to Southeast Asia. It is generally a childhood disease and our results are consistent with that trend because it was the commonest cause of AUFI among children [73–75]. In the past, dengue cases were mostly hospitalized irrespective of the severity of the disease. However, with the new admission criteria which includes clinical, laboratory, and dengue haemorrhage fever (DHF) predictive parameters [76], only severe cases of dengue: DHF and dengue shock syndrome (DSS) are admitted. The admission criteria were not clear in our study since most of the cases were from both OPD and IPD.

This review confirms that influenza is also an important cause of AUFI in the region, being the fourth commonest cause. Persistence of influenza virus especially in Southeast Asia is thought to be mediated by domestic ducks and large live poultry markets acting as a virus reservoir [77, 78]. Seasonal influenza is a highly transmissible, abrupt, and usually a self-limiting febrile infection of the respiratory tract and the majority of patients would present to outpatient departments [79, 80]. In many countries, the disease burden from influenza is underestimated because many cases are undiagnosed.

Typhoid fever was also identified as one of the major causes of AUFI. Previous reports have indicated that children are most at risk of developing typhoid fever [81]. The disease remains an important public health problem in developing countries. Similarly, rickettsial diseases including scrub typhus (*Orientia tsutsugamushi*) and murine typhus (*Rickettsia typhi*) were responsible for a small fraction of AUFI in this review. However, it is important to note that rickettsial diseases are an important cause of febrile illness worldwide but are often undiagnosed, sometimes leading to life-threatening conditions [82–85]. Given rickettsial infections are treatable causes of AUFI, greater recognition of scrub typhus and murine typhus is important to increase the index of suspicion amongst the physicians so that cases are not missed.

Protozoal infection particularly malaria was responsible for 3.7% of all AUFI cases. This figure is likely to have been an underestimate because four studies excluded malaria patients in their analysis as their inclusion criteria were non-malarial patients. Of the 11 member countries in the WHO SEA Region, 10 are endemic for malaria. Six countries (Bhutan, Democratic People’s Republic of Korea, Indonesia, Nepal, Sri Lanka, and Thailand) are aiming for malaria elimination as a longer-term goal [86], and Sri Lanka has already eliminated malaria [87].

We found that 1.0% of AUFIs were associated with co-infections, the majority being in inpatients. Patients not responding to treatment for a particular infection or those in whom the presentation was atypical or severe should be suspected of harbouring a second infectious agent. The possibility of co-infections of leptospirosis with hepatitis E virus (HEV) [88] has been described as water is the vehicle of transmission for both pathogens. The under-diagnosis of mixed infections is very likely due to the overlapping clinical spectrum [89]. The relatively high morbidity and mortality in mixed infections underscores the need for greater awareness of the possibility of mixed infections as well as the need for optimal use of microbiological laboratory services to reach a specific diagnosis [88].

Causes of fever remained unknown in more than half of patients with AUFI in this review. Similar findings have been reported in other studies, including a review of AUFI in South Asian countries [90]. A lack of an established diagnosis could be partly due to the fact that laboratory confirmations were not done in many studies of acute self-limiting viral infections. In addition, commercial serological rapid diagnostic tests used are semi-quantitative ELISAs that detect antibodies and are not conclusive of the present or past infection [91, 92]. For some pathogens, definitive diagnosis requires demonstration of a serial rise in antibody titres over a specific time period. Noncompliance of patients to report for repeat serological tests following improvement of the illness remains a major drawback in serology-based diagnostics [10, 93]. Moreover, ELISAs have poor specification and cross reactions are common [94]. Antigen-based or PCR-based diagnostics have been increasingly introduced to overcome these problems. However, their availability and affordability in resource-poor countries are limited, and the fact that they are not freely available in most government-run health institutions means that accessibility to such tests is limited to those in the private sector who can afford to pay from their own pocket [93].

This review has several limitations. Interpretation of data in this study should take into consideration the heterogeneity of the reviewed studies including study design, patient sampling and diagnostic testing. In addition, many of these studies were descriptive studies. Furthermore, there is no reliable way to judge the quality of heterogeneous descriptive studies included in this review. Some articles failed to report duration of fever and definition of AUFI varied widely between the studies. Aetiologies of AUFI of less than one-week duration would likely differ from those of a minimum of three weeks. Therefore, adherence to a common case definition between studies is important to make comparisons more reliable. Seasonal variation of diseases such as influenza, changes in disease patterns due to economic development, urbanization, environmental changes and changes in population densities during the last 15 years could have affected observed aetiologies and disease patterns. In addition, data from some countries including Bhutan and Timor-Leste were not available and results were also dominated by studies from India and Thailand. Since English is not the primary language in most of these countries, restricting the studies included in this review to studies published in English may have affected the findings.

Algorithms for the management of fevers at the community level as well as for inpatients have been developed by WHO [59]. A lack of knowledge of the geographical heterogeneity in AUFI aetiology prevents local adaptation of generic protocols, and thus precludes better targeting of drugs and implementation of early, effective management [95]. Therefore, it is necessary that data on pathogen presence collected incidentally in various studies and data collected by surveillance mechanisms be analysed systematically and mapped to provide information on the distribution and prevalence of infectious aetiologies of AFIs. Clinical algorithms could then be adapted, greatly improving targeting of treatment. Strengthening of notification systems (including sentinel systems) and sharing of data between clinical research communities will be important to construct more comprehensive information on geographically specific aetiologies of AUFI.

## Conclusion

In this study the most common causes of AUFI were viral, followed by bacterial and protozoal (malaria) infections. Dengue was the commonest virus that caused AUFI while leptospirosis and typhoid were important bacterial infectious causes. The challenges of unidentified causes of AUFI can be partly overcome by roll-out of affordable serological tests. It is imperative that data on pathogen presence collected incidentally in various studies and data collected by surveillance systems be analysed systematically and mapped to provide information on the distribution and prevalence of infectious aetiologies of AFIs for improving treatment and prevention programmes.

## Additional file

Additional file 1: **Table S1.** Inclusion and exclusion criteria. **Table S2.** Risk of bias questionnaire and scale. **Table S3.** Common presenting symptoms among patients. **Table S4.** Different diagnostic tools used in the studies. **Table S5.** Risk of bias scores of included studies. (DOCX 25 kb)

## Data Availability

The data used for this analysis can be available upon request to the corresponding author (KW).

## Abbreviations

AFI: acute febrile illness
AUFI: acute undifferentiated febrile illness
CFR: case fatality rate
DF: dengue fever
DHF: dengue haemorrhage fever
DSS: dengue shock syndrome
ED: emergency department
ELISA: enzyme-linked immunosorbent assay
HAV: Hepatitis A virus
HBV: Hepatitis B virus
HEV: Hepatitis E virus
IP: inpatients
JE: Japanese encephalitis
MAT: microscopic agglutination test
OPD: outpatient department
PCR: polymerase chain reaction
SA: South Asia
SEA: Southeast Asia
UAG: unspecified age group
WHO: World Health Organization
