# Supplementary material for: Diversity of infectious aetiologies of acute undifferentiated febrile illnesses in south and Southeast Asia: a systematic review
Source: BMC Infect Dis. 2019 Jul 4;19:577. doi: 10.1186/s12879-019-4185-y (PMC6610835; doi:10.1186/s12879-019-4185-y)
Supplement: Supplementary file 1 — Table S1. Inclusion and exclusion criteria. Table S2. Risk of bias questionnaire and scale. Table S3. Common presenting symptoms among patients. Table S4. Different diagnostic tools used in the studies. Table S5. Risk of bias scores of included studies. (DOCX 25 kb) [file 12879_2019_4185_MOESM1_ESM.docx]

**Additional file 1**

**Table S1 Inclusion and exclusion criteria.**

| Inclusion criteria | |
| --- | --- |
|  | Prevalence studies in a sample of consecutive febrile patients attending health facilities |
|  | Incidence studies with active and passive case detection |
|  | Studies done in tropical and subtropical south and southeast Asian countries |
|  | All age groups |
|  | Study conducted on out-patients, inward patients or community based |
|  | Laboratory analysis conducted (serology, culture) |
| Exclusion criteria | |
|  | Studies conducted between 1998 and 2019 |
|  | Editorials/ opinions rather than publication of new data |
|  | Vaccine and drug trials |
|  | Case reports |
|  | Post travel illness (acute febrile illnesses associated with travel) |
|  | Patients included with other causes of fever (i.e. signs of localized infections at commencement of the study) |
|  | Studies done in Middle-Eastern Countries |
|  | Non-human studies |

**Table S2 Risk of bias questionnaire and scale.**

| **Item** | **Questions** | **Score** |
| --- | --- | --- |
|  |  | 1=Yes/Not applicable, 0=No/Unclear |
|  | **External validity** |  |
| 1. | Was the study's target population a close representation of the national population in relation to relevant variables? |  |
| 2. | Was some form of random selection used to select the sample, OR was a census undertaken? |  |
| 3. | Was the likelihood of nonresponse bias minimal? |  |
|  | **Internal validity** |  |
| 4. | Were data collected directly from the subjects (as opposed to a proxy)? |  |
| 5. | Was an acceptable case definition used in the study? |  |
| 6. | Was the study instrument that measured the parameter of interest shown to have validity and reliability? |  |
| 7. | Was the same mode of data collection used for all subjects? |  |
| 8. | Was the length of the shortest prevalence period for the parameter of interest appropriate? |  |

**Table S3 Common presenting symptoms among patients.**

| **Symptom** | **Number (30,397)** | **Percent** |
| --- | --- | --- |
| **Headache** | 12,072 | 39.7 |
| **Myalgia** | 4,757 | 15.6 |
| **Arthralgia** | 4,190 | 13.8 |
| **Chills** | 6,241 | 20.5 |
| **Vomiting** | 2,319 | 7.6 |
| **Diarrhoea** | 2,524 | 8.3 |
| **Cough** | 9,035 | 29.7 |
| **Rash** | 1,391 | 4.6 |
| **Abdominal pain** | 2,074 | 6.8 |
| **Constipation** | 496 | 1.6 |

**Table S4 Different diagnostic tools used in the studies.**

| **Sl. No** | **Authors** | **Tests** |
| --- | --- | --- |
| 1 | Abhilash et al., 2016 | Microscopy, Serology (IgM, IgG), Blood Culture |
| 2 | Ahmad et al., 2016 | Microscopy, Serology (IgM, IgG) |
| 3 | Andrews et al., 2014 | Serology, Blood Culture |
| 4 | Arora et al., 2017 | Microscopy, Serology |
| 5 | Capeding et al., 2013 | Serology |
| 6 | Chheng et al., 2013 | Serology, Blood Culture, Molecular |
| 7 | Chikkaveeraiah et al., 2016 | Microscopy, Serology, Blood Culture |
| 8 | Cripsal et al., 2010 | Serology |
| 9 | Das et al., 2015 | Serology, Blood Culture |
| 10 | Ellis et al., 2006 | Microscopy, Serology, Molecular |
| 11 | Gopalakrishnan et al., 2013 | Microscopy, Serology, Blood Culture |
| 12 | Joshi et al., 2008 | Microscopy, Serology, Blood Culture |
| 13 | Kammili et al., 2013 | Serology |
| 14 | Kashinkuti et al., 2013 | Microscopy, Serology, Blood Culture |
| 15 | Kasper et al., 2012 | Microscopy, Serology, Blood Culture, Molecular |
| 16 | Kumar et al., 2008 | Serology, Molecular |
| 17 | Laoprasopwattana et al., 2012 | Serology, Molecular |
| 18 | Leelarasamee et al., 2004 | Serology, Blood culture |
| 19 | Mayxay et al., 2013 | Microscopy, Serology, Blood Culture, Molecular |
| 20 | McGready et al., 2010 | Microscopy, Serology, Blood Culture, Molecular |
| 21 | Mittal et al., 2015 | Microscopy, Serology |
| 22 | Murdoch et al., 2004 | Microscopy, Serology, Blood Culture |
| 23 | Oishr et al., 2006 | Serology, Molecular |
| 24 | Phuong et al., 2006 | Serology |
| 25 | Pradutkanchana et al., 2003 | Serology |
| 26 | Punjabi et al., 2012 | Microscopy, Serology, Blood Culture |
| 27 | Rafizah et al., 2012 | Serology |
| 28 | Rani et al., 2016 | Serology |
| 29 | Ray et al., 2012 | Serology, Molecular, Sequencing |
| 30 | Reller et al., 2011 | Serology |
| 31 | Reller et al., 2012 | Serology, Molecular |
| 32 | Sabchareon et al., 2012 | Serology, Molecular |
| 33 | Suttinont et al., 2006 | Serology |
| 34 | Thompson et al., 2015 | Serology, Molecular |
| 35 | Zaki et al., 2010 | Serology |
| 36 | Kingston et al., 2018 | Molecular |
| 37 | Raina et al., 2018 | Microscopy, Serology, Blood Culture |
| 38 | Shelke et al., 2017 | Serology |
| 39 | Gautam et al., 2019 | Serology |
| 40 | Bodinayake et al., 2018 | Serology, Molecular, Sequencing |
| 41 | Salagre et al., 2017 | Microscopy, Serology, Molecular |
| 42 | Andrews et al., 2018 |  |
| 43 | Wangrangsimakul et al., 2018 | Serology, Blood Culture, Molecular |

**Table S5 Risk of bias scores of included studies**

| **Sl. No** | **Study** | **1** | **2** | **3** | **4** | **5** | **6** | **7** | **8** | **Score** |
| --- | --- | --- | --- | --- | --- | --- | --- | --- | --- | --- |
| 1 | Abhilash et al., 2016 | 0 | 1 | 1 | 1 | 1 | 1 | 1 | 1 | 7 |
| 2 | Ahmad et al., 2016 | 1 | 1 | 1 | 1 | 1 | 1 | 1 | 1 | 8 |
| 3 | Andrews et al., 2014 | 0 | 1 | 1 | 1 | 1 | 1 | 0 | 1 | 6 |
| 4 | Arora et al., 2017 | 0 | 0 | 1 | 1 | 0 | 1 | 1 | 0 | 4 |
| 5 | Capeding et al., 2013 | 1 | 1 | 1 | 1 | 1 | 1 | 0 | 1 | 7 |
| 6 | Chheng et al., 2013 | 0 | 1 | 1 | 1 | 1 | 1 | 1 | 1 | 7 |
| 7 | Chikkaveeraiah et al., 2016 | 0 | 1 | 1 | 1 | 1 | 1 | 1 | 1 | 7 |
| 8 | Cripsal et al., 2010 | 0 | 1 | 1 | 1 | 1 | 1 | 1 | 1 | 7 |
| 9 | Das et al., 2015 | 1 | 0 | 1 | 1 | 1 | 1 | 1 | 1 | 7 |
| 10 | Ellis et al., 2006 | 0 | 1 | 0 | 1 | 1 | 1 | 1 | 1 | 6 |
| 11 | GopalaKrishnan et al., 2013 | 0 | 0 | 1 | 1 | 1 | 1 | 1 | 1 | 6 |
| 12 | Joshi et al., 2008 | 0 | 1 | 1 | 0 | 1 | 1 | 1 | 0 | 5 |
| 13 | Kammili et al., 2013 | 1 | 0 | 1 | 1 | 1 | 1 | 1 | 1 | 7 |
| 14 | Kashinkuti et al., 2013 | 0 | 0 | 1 | 1 | 0 | 1 | 1 | 1 | 5 |
| 15 | Kasper et al., 2012 | 1 | 0 | 1 | 1 | 1 | 1 | 1 | 1 | 7 |
| 16 | Kumar et al., 2008 | 0 | 1 | 1 | 1 | 1 | 1 | 1 | 1 | 7 |
| 17 | Laoprasopwattana et al., 2012 | 0 | 1 | 0 | 1 | 1 | 1 | 1 | 1 | 6 |
| 18 | Leelarasamee et al., 2004 | 0 | 1 | 0 | 1 | 1 | 1 | 1 | 1 | 6 |
| 19 | Mayxay et al., 2013 | 0 | 1 | 1 | 1 | 1 | 1 | 1 | 1 | 7 |
| 20 | McGready et al., 2010 | 0 | 0 | 1 | 1 | 1 | 1 | 1 | 0 | 5 |
| 21 | Mittal et al., 2015 | 0 | 1 | 0 | 0 | 1 | 1 | 1 | 1 | 5 |
| 22 | Murdoch et al., 2004 | 0 | 1 | 1 | 1 | 1 | 1 | 1 | 1 | 7 |
| 23 | Oishr et al., 2006 | 0 | 0 | 1 | 0 | 1 | 1 | 1 | 0 | 4 |
| 24 | Phuonr et al., 2006 | 1 | 1 | 1 | 1 | 1 | 1 | 1 | 1 | 8 |
| 25 | Pradutkanchana et al., 2003 | 0 | 0 | 0 | 1 | 1 | 1 | 1 | 1 | 5 |
| 26 | Punjabi et al., 2012 | 0 | 0 | 0 | 1 | 1 | 1 | 1 | 1 | 5 |
| 27 | Rafizah et al., 2012 | 0 | 0 | 1 | 1 | 0 | 1 | 1 | 1 | 5 |
| 28 | Rani et al., 2016 | 1 | 0 | 0 | 0 | 0 | 1 | 1 | 1 | 4 |
| 29 | Ray et al., 2012 | 1 | 1 | 1 | 1 | 1 | 1 | 1 | 1 | 8 |
| 30 | Reller et al., 2011 | 1 | 1 | 1 | 1 | 1 | 1 | 1 | 1 | 8 |
| 31 | Reller et al., 2012 | 1 | 0 | 0 | 1 | 1 | 1 | 1 | 1 | 6 |
| 32 | Sabchareon et al., 2012 | 0 | 1 | 0 | 1 | 0 | 1 | 1 | 1 | 5 |
| 33 | Suttinont et al., 2006 | 0 | 0 | 1 | 1 | 0 | 1 | 1 | 1 | 5 |
| 34 | Thompson et al., 2015 | 1 | 1 | 0 | 1 | 0 | 1 | 1 | 1 | 6 |
| 35 | Zaki et al., 2010 | 0 | 1 | 1 | 1 | 1 | 1 | 1 | 1 | 7 |
| 36 | Kingston et al., 2018 | 0 | 0 | 1 | 1 | 0 | 1 | 1 | 1 | 5 |
| 37 | Raina et al., 2018 | 0 | 0 | 1 | 1 | 0 | 1 | 1 | 0 | 4 |
| 38 | Shelke et al., 2017 | 1 | 1 | 1 | 1 | 0 | 1 | 1 | 1 | 7 |
| 39 | Gautam et al., 2019 | 1 | 0 | 1 | 1 | 0 | 1 | 1 | 1 | 6 |
| 40 | Bodinayake et al., 2018 | 1 | 0 | 0 | 1 | 1 | 1 | 1 | 1 | 6 |
| 41 | Salagre et al., 2017 | 1 | 0 | 1 | 1 | 0 | 1 | 1 | 1 | 6 |
| 42 | Andrews et al., 2018 | 0 | 0 | 1 | 1 | 1 | 1 | 1 | 1 | 6 |
| 43 | Wangrangsimakul et al., 2018 | 0 | 0 | 1 | 1 | 1 | 1 | 1 | 1 | 6 |
|  | **Total** | **15** | **22** | **32** | **39** | **31** | **43** | **41** | **38** |  |
